# Abnormal changes of bone metabolism markers with age in children with cerebral palsy

**DOI:** 10.3389/fped.2023.1214608

**Published:** 2023-08-01

**Authors:** Wen Xing, Lin Liang, Na Dong, Liang Chen, Zhizhong Liu

**Affiliations:** Department of Clinical Laboratory, Beijing Bo'ai Hospital, China Rehabilitation Research Center, Beijing, China

**Keywords:** cerebral palsy, bone metabolism, bone formation, bone resorption, phosphorus-calcium metabolism

## Abstract

Cerebral palsy (CP) is a broad range of diseases with permanent and nonprogressive motor impairments, carrying a high cost for both the individual and the society. The characteristics of low bone mineral density and high risk of fractures suggest that bone metabolism disorders are present in CP. This study aims to investigate the association between indicators of bone metabolism and children with CP. A total of 139 children (75 children with CP and 64 healthy controls) were included in this cross-sectional study. Participants were divided into three age groups (0–2 years, 2.1–4 years, and 4.1–7 years). All children with CP were diagnosed according to clinical criteria and furtherly divided into clinical subtypes. The levels of total procollagen type I N-terminal propeptide (TPINP), N-MID osteocalcin (OC), beta-crosslaps (β-CTX), 25-hydroxyvitamin D (25-OHD) and parathyroid hormone (PTH) in the serum were measured with corresponding detection kits according to the manufacturer's instructions. Serum levels of TPINP and 25-OHD were lower with older age, whereas β-CTX and PTH were higher with older age. In the CP group, TPINP (age 0–2 years and 2.1–4 years) and OC (age 2.1–4 years) levels were higher, while β-CTX (age 2.1–4 years and 4.1–7 years) and PTH (age 2.1–4 years) values were lower than the control group. In addition, there were no statistically significant differences in the levels of these indicators among the CP subgroups with different clinical characteristics. Our study shows that bone turnover markers, indicators of bone metabolism, in children with CP differ significantly from healthy controls. The indicators we studied changed with age, and they did not correlate with disease severity.

## Introduction

Cerebral palsy (CP) is the most prevalent physical disability in childhood, with a prevalence around 2 per 1,000 live births (1). CP is a heterogeneous group of disorders of movement and posture attributed to a defect or lesion in the developing fetal or infant brain (2). Although the etiology of CP is multifactorial and complex, the exact causative mechanisms contributing to pathogenesis remain unknown, making progress towards prevention and treatment strategies difficult (3).

The skeleton is an important component of vertebrates’ body, providing structural support for locomotion and protection for major organs (4). Meanwhile, bone tissue also functions as an endocrine organ (5). Bone cells in the human body are continuously involved in cellular metabolism, a dynamic process of osteoblastic bone formation and osteoclastic bone resorption (6). Studies on different populations (older adults, menopausal females, etc.) reports that bone metabolism changes are closely related to bone density and fractures (7–10), but this is less studied in children and especially in children with CP. Importantly, low bone mineral density is common in children with CP (8). Therefore, further investigation is required to determine the attributes of bone metabolism in children with CP.

TPINP, OC, β-CTX, 25-OHD and PTH are commonly used as important clinical indicators of bone metabolism. Of these, TPINP and OC are measured as indicators of bone formation, β-CTX can be used as an indicator of bone resorption. The other two metrics, 25-OHD and PTH, play central roles in regulating calcium-phosphate metabolism (11–14). These indicators can assist in disease diagnosis (15), treatment monitoring (16, 17), predicting changes in bone density (16), and predicting fracture risk (18) in populations with different physiological or pathological states (19). The skeletal expansion undergoes dramatic changes as humans develop from infancy to adulthood, especially in early childhood and puberty. Previous studies reported that serum levels of bone metabolism markers reflect skeletal development during puberty (20). As skeletal development is nonlinear during puberty, results of bone turnover markers must be interpreted in combination with the subject's maturation stage. To date, there is little research on differences in these indicators of bone metabolism by age in children with CP as compared to children without CP. As there are rapid changes in skeletal growth, knowledge of bone metabolism by age in children with CP may inform on biological aspects of poor bone health associated with CP.

The objective of this study was to test for differences in serum indicators of bone metabolism by age between children with and without CP.

## Materials and methods

### Subjects

A total of 139 children (75 children with CP and 64 typically developing children) were included in the present study. All the children with CP were enrolled from Beijing Bo’ ai Hospital, China Rehabilitation Research Center. Clinical data were obtained from all cases, including gestational age, gender, details of pregnancy and birth, head circumference, birth weight, Apgar scores, neonatal events, and height and weight at the time of the assessment. All patients were diagnosed according to the clinical criteria and classified into spastic (furtherly divided into hemiplegia, diplegia and quadriplegia), ataxic, dyskinetic subtypes on the basis of the guidelines proposed by the Surveillance of CP in Europe (SCPE) (21). Gross motor impairment was assessed by Gross Motor Function Classification System (GMFCS) (22), which ranges from level I to level V. Children with CP classified as GMFCS level I–III have minimal-moderate motor impairment and activity limitation. Children with CP classified as levels IV and V correspond with having no independent walking ability. Medical complications associated with CP were also recorded (e.g., cognitive impairment, epilepsy, feeding difficulties). Exclusion criteria included children with myopathy, hypotonia, neural tube defect, ataxia, chromosomal anomaly, genetic syndrome, or other chronic disease that could influence bone metabolism. Healthy children that did not have any history of neurological disorders and mental disorders were recruited from Maternal & Child Health Hospital in Fengtai and Xicheng Districts. The study was approved by the Ethics Committee of Beijing Bo’ai Hospital, China Rehabilitation Research Center.

### Samples

Peripheral whole blood samples were drawn in procoagulant tubes from the median cubital vein after an eight hour fast and taken between 8:00 and 10:00 am to avoid diurnal variation. Then, the blood samples were centrifuged at 4,000 rpm for 10 min and aliquots of serum were immediately stored at −80°C until analysis. None of the samples used in the study showed hemolysis.

### Analytical determinations

The levels of TPINP, OC, β-CTX, 25-OHD and PTH in the serum were measured with corresponding detection kits according to the manufacturer's instructions (Roche Diagnostics GmbH, Germany). Analysis was performed using the Roche Cobas e411 Automatic Electrochemiluminescence Immunoassay Instrument.

### Statistical analysis

The statistical analyses were performed using GraphPad Prism (Version 7.00, GraphPad Software Inc., USA). Age was stratified into three groups: 0–2.0, 2.1–4.0, and 4.1–7 years old. The indicators of bone metabolism were presented as median [interquartile range (IQR)]. The differences in the distribution of clinical characteristics between the CP group and healthy controls were evaluated by Chi-Square test or Fisher's exact test for categorical variables (age, gender). The comparisons between the subgroups in the CP group were statistically evaluated by Student's *t*-test or Mann-Whitney *U*-test. The comparisons within groups were analyzed with a nonparametric Kruskal-Wallis test with a *post hoc* Dunn's test. A *p*-value <0.05 was considered statistically significant.

### Exploratory analysis

The relationship between the primary bone resorption indicator, β-CTX, with the primary bone formation indicators, TPINP and OC, for both cohorts including all ages 0–7 years old was examined using simple linear regression. The regression equation, Pearson correlation coefficient, and *p*-value were reported for each cohort. Regression analyses were performed before and after excluding outliers, which was determined by values that were ≥3 standard deviations above the group mean. To assess for the possibility of de-coupling of bone resorption and formation between cohorts, the variance analysis of factorial design examined the main and interaction effects for group (CP vs. controls) and β-CTX for each bone formation indicator before and after adjusting for age (as continuous). The interaction effect was the primary focus of this analysis.

## Results

### Baseline characteristics

Descriptive characteristics of children with CP (*n* = 75) and healthy controls (*n* = 64) are presented in Table 1. Notably, there were no significant differences between groups for gender for the full cohorts (*p *= 0.386) or for continuous age within each age category (*p *> 0.120) (Table 1).

**Table 1:** Baseline characteristics of CP patients and healthy controls.

Clinical characteristics	CP (*n* = 75)	Controls (*n* = 64)	*p*-value
Age in years			
0–2	1.10 ± 0.364 27 (36.0%)	1.140 ± 0.232 9 (14.1%)	0.741
2.1–4	2.75 ± 0.558 28 (37.3%)	2.97 ± 0.485 31 (48.4%)	0.124
4.1–7	5.13 ± 0.868 20 (26.7%)	5.05 ± 0.831 24 (37.5%)	0.769
Gender, *n* (%)			
Male, 0–7.0 years	49 (65.3%)	37 (57.8%)	0.386
0–2 years	16 (21.3%)	7 (10.9%)	
2.1–4 years	19 (25.3%)	16 (25.0%)	
4.1–7 years	14 (18.7%)	14 (21.9%)	
Female, 0–7.0 years	26 (34.7%)	27 (42.2%)	
0–2 years	11 (14.7%)	2 (3.1%)	
2.1–4 years	9 (12.0%)	15 (23.4%)	
4.1–7 years	6 (8.0%)	10 (15.6%)	
Height			
0–2 years	76.50 ± 7.023		
2.1–4 years	91.30 ± 6.941		
4.1–7 years	108.30 ± 8.509		
Weight			
0–2 years	9.87 ± 1.644		
2.1–4 years	13.61 ± 2.790		
4.1–7 years	19.33 ± 4.222		
CP Subtypes, *n* (%)			
Spastic hemiplegia, 0–7.0 years	16 (21.3%)		
0–2 years	7 (9.3%)		
2.1–4 years	8 (10.7%)		
4.1–7 years	1 (1.3%)		
Spastic diplegia, 0–7.0 years	32 (42.7%)		
0–2 years	8 (10.7%)		
2.1–4 years	14 (18.7%)		
4.1–7 years	10 (13.3%)		
Spastic quadriplegia, 0–7.0 years	9 (12.0%)		
0–2 years	4 (5.3%)		
2.1–4 years	1 (1.3%)		
4.1–7 years	4 (5.3%)		
Ataxic, 0–7.0 years	2 (2.7%)		
0–2 years	0 (0%)		
2.1–4 years	0 (0%)		
4.1–7 years	2 (2.7%)		
Dyskinetic, 0–7.0 years	9 (12.0%)		
0–2 years	5 (6.7%)		
2.1–4 years	2 (2.7%)		
4.1–7 years	2 (2.7%)		
Non-Classified, 0–7.0 years	7 (9.3%)		
0–2 years	3 (4.0%)		
2.1–4 years	3 (4.0%)		
4.1–7 years	1 (1.3%)		
GMFCS level, *n* (%)			
I, 0–7.0 years	12 (16.0%)		
0–2 years	1 (1.3%)		
2.1–4 years	7 (9.3%)		
4.1–7 years	4 (5.3%)		
II, 0–7.0 years	19 (25.3%)		
0–2 years	7 (9.3%)		
2.1–4 years	8 (10.7%)		
4.1–7 years	4 (5.3%)		
III, 0–7.0 years	19 (25.3%)		
0–2 years	5 (6.7%)		
2.1–4 years	6 (8.0%)		
4.1–7 years	8 (10.7%)		
IV, 0–7.0 years	14 (18.7%)		
0–2 years	7 (9.3%)		
2.1–4 years	6 (8.0%)		
4.1–7 years	1 (1.3%)		
V, 0–7.0 years	11 (14.7%)		
0–2 years	7 (9.3%)		
2.1–4 years	1 (1.3%)		
4.1–7 years	3 (4.0%)		
Complications, *n* (%)			
Cortical visual impairment, 0–7.0 years	12 (16.0%)		
0–2 years	2 (2.7%)		
2.1–4 years	5 (6.7%)		
4.1–7 years	5 (6.7%)		
Sensorineural auditory impairment, 0–7.0 years	1 (1.3%)		
0–2 years	1 (1.3%)		
2.1–4 years	0 (0%)		
4.1–7 years	0 (0%)		
Communication difficulties, 0–7.0 years	25 (33.3%)		
0–2 years	7 (9.3%)		
2.1–4 years	10 (13.3%)		
4.1–7 years	8 (10.7%)		
Cognitive impairment, 0–7.0 years	17 (22.7%)		
0–2 years	5 (6.7%)		
2.1–4 years	5 (6.7%)		
4.1–7 years	7 (9.3%)		
Feeding difficulties, 0–7.0 years	1 (1.3%)		
0–2 years	0 (0%)		
2.1–4 years	1 (1.3%)		
4.1–7 years	0(%)		
Epilepsy, 0–7.0 years	11 (14.7%)		
0–2	4 (5.3%)		
2.1–4	3 (4.0%)		
4.1–7	4 (5.3%)		
Anti-convulsant treatments, 0–7.0 years	15 (20.0%)		
0–2 years	5 (6.7%)		
2.1–4 years	6 (8.0%)		
4.1–7 years	4 (5.3%)		

Data are shown as *n* (%) or mean ± SD.

CP, cerebral palsy; GMFCS, Gross Motor Function Classification System.

### Comparison of different indicators of bone metabolism

**Figure 1:**
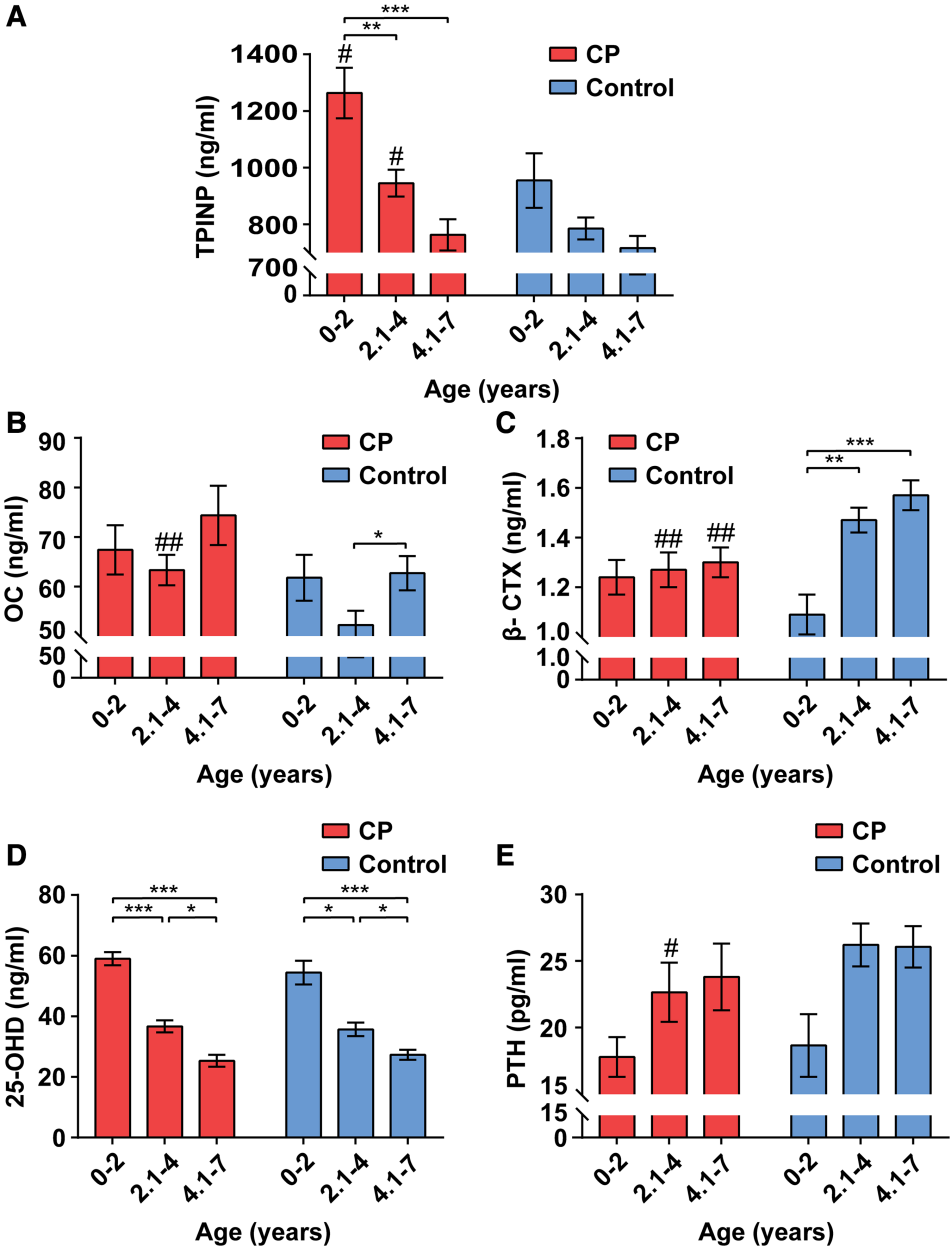
Alterations of bone metabolism in CP group and healthy control group by age ranges. The values of total procollagen type I N-terminal propeptide (TPINP) (**A**), N-MID osteocalcin (OC) (**B**), beta-crosslaps (β-CTX) (**C**), 25-hydroxyvitamin D (25-OHD) (**D**) and parathyroid hormone (PTH) (**E**) in different groups. Data represent the Median (1st, 3rd quartiles). # and * indicate inter-group and intra-group comparison results, respectively. *p < 0.05; **p < 0.01; ***p < 0.001; ^#^*p* < 0.05; ^##^*p* < 0.01.

The serum indicators of bone metabolism for children with CP and healthy controls by age group are presented in Figure 1. In general, and for both the CP and healthy control groups, TPINP and 25-OHD levels were lower with older age groups, PTH and β-CTX were higher with older age groups, and OC showed a bimodal pattern with age groups.

Among 0–2-year-olds, children with CP had higher serum levels of TPINP (*p *= 0.037) compared to healthy controls. Among 2.1–4-year-olds, children with CP had higher serum levels of TPINP (*p *= 0.015) and OC (*p *= 0.003) and lower levels of serum β-CTX (*p *= 0.004) and PTH (*p *= 0.032) compared to healthy controls. Among 4.1–7-year-olds, children with CP had lower serum levels β-CTX (*p *= 0.002) compared to healthy controls.

### Association between different markers and clinical characteristics of CP

Due to a limited sample size, spastic and non-spastic (ataxic, dyskinetic, non-classified) types of CP were grouped. There were no statistical differences in the indicators of bone metabolism after stratifying the CP cohort by spastic (*n* = 57) or non-spastic (*n* = 18) type (Table 2). The descriptive characteristics of the spastic and non-spastic type CP groups are presented in Supplementary Table S1. Notably, age was similar (2.53 ± 1.59 vs. 2.27 ± 1.67, *p *= 0.387) but the spastic type CP group had a higher proportion of males (71.9% vs. 44.4%, *p *= 0.047).

**Table 2:** Association between indicators and subtypes in CP group.

	Spastic (*n* = 57)	Non-Spastic (*n* = 18)	*p-*value
TPINP	1,062.00 (766.90–1,200.00)	1,015.00 (653.70–1,200.00)	0.384
OC	66.09 (53.75–78.02)	60.54 (48.04–73.78)	0.369
β-CTX	1.27 (1.05–1.44)	1.14 (0.95–1.50)	0.394
25-OHD	39.26 (27.53–56.78)	38.57 (25.54–60.19)	0.980
PTH	18.57 (13.39–28.15)	18.31 (15.9–24.01)	0.997

Data are shown as median (interquartile range, IQR) and *n*.

TPINP, total procollagen type I N-terminal propeptide; OC, N-MID osteocalcin; β-CTX, beta-crosslaps; 25-OHD, 25-hydroxyvitamin D; PTH, parathyroid hormone.

Due to a limited sample size, GMFCS was stratified by I-III and IV/V. There were no statistical differences in the indicators of bone metabolism after stratifying the CP cohort by GMFCS I-III (*n* = 50) or GMFCS IV/V (*n* = 25) type (Table 3). The descriptive characteristics of the GMFCS I-III and GMFCS IV/V groups are presented in Supplementary Table S2. Notably, the GMFCS I-III group was older on average (2.81 ± 1.65 vs.1.78 ± 1.26, *p *= 0.040) but there were no differences in gender (male, 68.0% vs. 60.0%).

**Table 3:** Association between indicators and GMFCS level in CP group.

	GMFCS(I-III) (*n* = 50)	GMFCS(IV-V) (*n* = 25)	*p-*value
TPINP	1,031.00 (719.40–1,200.00)	1,113.00 (726.70–1,200.00)	0.568
OC	66.92 (54.64–79.03)	56.72 (48.04–67.49)	0.105
β-CTX	1.28 (1.06–1.44)	1.14 (0.95–01.38)	0.215
25-OHD	38.59 (24.36–54.98)	42.85 (29.04–62.04)	0.261
PTH	18.43 (13.92–27.73)	18.31 (12.90–24.72)	0.483

Data are shown as median (interquartile range, IQR) and *n*.

GMFCS, Gross Motor Function Classification System; TPINP, total procollagen type I N-terminal propeptide; OC, N-MID osteocalcin; β-CTX, beta-crosslaps; 25-OHD, 25-hydroxyvitamin D; PTH, parathyroid hormone.

The spastic CP was further subdivided into hemiplegia, diplegia and quadriplegia. The basic clinical information is shown in Supplementary Table S3. There were no significant differences in age (2.03 ± 1.37 vs. 2.72 ± 1.57 vs. 2.73 ± 1.98, *p *= 0.290) and gender (*p *= 0.490) between the groups. We did not find any significant differences in variances among groups (Table 4).

**Table 4:** Association between indicators and spastic subtypes in CP group.

	Spastic subtypes	*N*	Median (25%–75% percentile)	Adjusted *p-*value
TPINP	Hemiplegia	16	1,139.00 (828.98–1,200.00)	
	Diplegia	32	1,048.50 (680.63–1,200.00)	
	Quadriplegia	9	984.20 (703.05–1,470.60)	
	Hemiplegia vs. Diplegia			0.824
	Hemiplegia vs. Quadriplegia			>0.999
	Diplegia vs. Quadriplegia			>0.999
OC	Hemiplegia	16	63.54 (56.89–76.81)	
	Diplegia	32	66.50 (50.94–77.24)	
	Quadriplegia	9	72.08 (52.44–94.97)	
	Hemiplegia vs. Diplegia			>0.999
	Hemiplegia vs. Quadriplegia			>0.999
	Diplegia vs. Quadriplegia			>0.999
β-CTX	Hemiplegia	16	1.26 (0.94–1.40)	
	Diplegia	32	1.20 (1.01–1.44)	
	Quadriplegia	9	1.39 (1.27–1.64)	
	Hemiplegia vs. Diplegia			>0.999
	Hemiplegia vs. Quadriplegia			0.123
	Diplegia vs. Quadriplegia			0.203
25-OHD	Hemiplegia	16	42.81 (29.71–62.07)	
	Diplegia	32	39.48 (25.52–55.15)	
	Quadriplegia	9	31.00 (21.01–53.45)	
	Hemiplegia vs. Diplegia			>0.999
	Hemiplegia vs. Quadriplegia			0.484
	Diplegia vs. Quadriplegia			>0.999
PTH	Hemiplegia	16	18.42 (12.46–31.63)	
	Diplegia	32	17.64 (13.25–28.04)	
	Quadriplegia	9	21.09 (17.69–27.98)	
	Hemiplegia vs. Diplegia			>0.999
	Hemiplegia vs. Quadriplegia			>0.999
	Diplegia vs. Quadriplegia			>0.999

Data are shown as median (interquartile range, IQR) and *n*.

TPINP, total procollagen type I N-terminal propeptide; OC, N-MID osteocalcin; β-CTX, beta-crosslaps; 25-OHD, 25-hydroxyvitamin D; PTH, parathyroid hormone.

### Exploratory analysis

**Figure 2:**
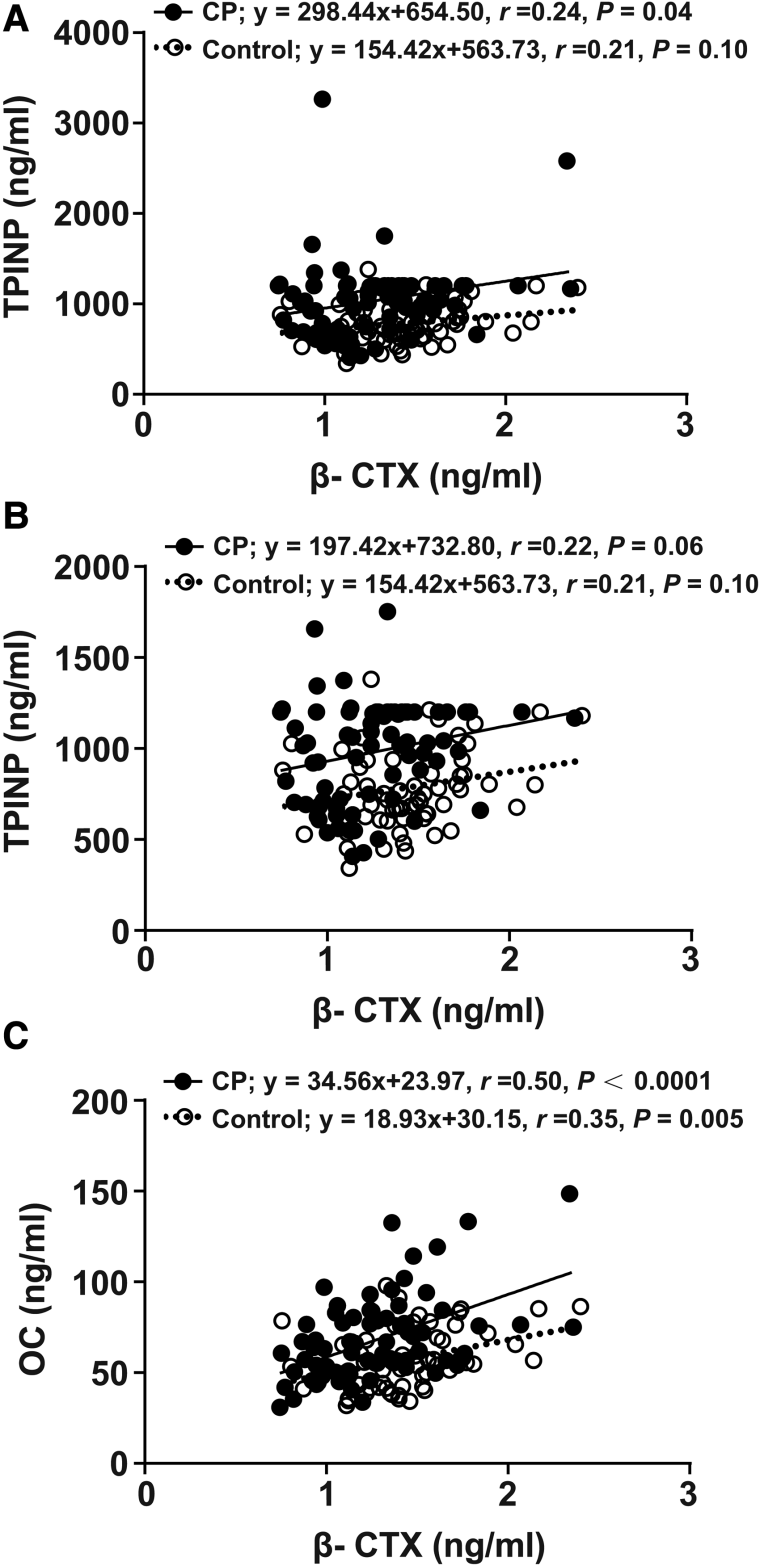
Linear regression analyses of indicators between the CP group and the control group. β-CTX regressed with TPINP (**A**), β-CTX regressed with TPINP (2 significant outliers were removed) (**B**), β-CTX regressed with OC (**C**). β-CTX, beta-crosslaps; TPINP, total procollagen type I N-terminal propeptide; OC, N-MID osteocalcin; CP, Cerebral palsy.

The association between β-CTX and TPINP and between β-CTX and OC is presented in Figure 2. There was evidence of 2 outliers for the TPINP values in the CP group. There was a positive, weak relationship between β-CTX and TPINP in the CP and controls groups, before and after excluding the outliers. The relationship was also positive and stronger between β-CTX and OC for both groups, especially in CP. There was no strong evidence of a group by β-CTX interaction for TPINP after excluding the 2 outliers before and after adjusting for age (*p* for interaction, 0.683 and 0.299, respectively). There was no evidence of a group by β-CTX interaction for OC before and after adjusting for age (*p* for interaction, 0.766 and 0.807).

## Discussion

In this study, we found significant differences in certain indicators of bone metabolism at specific age groups in children with CP when compared to a cohort of healthy controls. There was no evidence that the indicators of bone metabolism were mainly driven by clinical characteristics of CP, including the type of CP (e.g., spastic vs. non-spastic) or the severity of motor important via GMFCS.

In this study, we selected two indicators reflecting bone formation, including TPINP and OC. Type I procollagen, an osteoblast product, can form Type I collagen via enzymatic cleavage the pro-peptide (23). During the process, N-terminal propeptide, also referred to as TPINP, is released into circulation (24). This process corresponds to the formation of bone matrix (25). Thus, the level of TPINP is considered a reliable marker of bone formation. It is stable and no circadian rhythm (26). In this study, we found that TPINP concentrations gradually decreases with age (in the age band 0–7 years) in the healthy control groups, which is consistent with previous studies (27, 28). Meanwhile, our results showed that children with CP had higher serum levels TPINP than healthy controls at ages 0–4 years old, indicating higher levels of bone formation. As prior research has reported that children with CP have low bone density (29) and notable defects in bone size, shape or structure (30–32), we speculate that the higher bone formation in 0–4 year olds with CP is ineffective and even harmful, such as heterotopic ossification (33, 34). In addition, children with CP have increased fracture risk associated with low bone mineral density and high bone fragility (35). Fracture healing is a process of bone remodeling that is often accompanied by a significant enhancement of bone formation (36, 37).

OC is one of the most abundant non-collagenous proteins in bone tissue, which is secreted by osteoblasts (38). OC is partly involved in bone matrix formation and partly secreted into the peripheral blood (39, 40). Therefore, serum OC levels are used as a valuable indicator of bone formation (41). Although the circadian rhythm plays a role in serum concentrations of OC (42), blood was taken from participants around the same time during the day, thus mitigating bias in group differences due to the timing of blood draw. In this study, the bimodal pattern of serum levels of OC by age group is similar to that of a previous study (28). Further, among 2.1–4-year-olds, children with CP had higher levels of OC than healthy controls, which is consistent with the higher levels of the bone formation indicator, TPINP.

β-CTX is known as a marker of bone resorption, which reflects the degree of bone matrix degradation (43). Mature type I collagen of bone matrix degrades into β-CTX and releases into the bloodstream in the process of bone metabolism (44, 45). Our results showed a gain in bone resorption with age in the control groups. This may promote new bone formation, as bone resorption is a source of bone formation-stimulating factors (46). We found lower levels of β-CTX in the group with CP among 2.1–7-year-olds, which is not conducive to bone formation. The reasons may be due to exercise restriction and decrease in muscle stimulation (47).

The above data, taken together, suggest that bone formation in children with CP was higher than that in healthy children in different age groups. On the contrary, children with CP had lower bone resorption than healthy children with older age, particularly after age 2. Skeletal growth is the results of a dynamic balance between bone formation and bone resorption (48, 49), and this balance has been demonstrated to be disrupted in many pediatric diseases (50). In our study, imbalance in bone metabolism was also found in disease group, potentially causing growth restriction to some extent. These features deserve the physicians’ attention in the future. Some cytokines have multiple effects on RANKL-RANK/osteoprotegerin and WNT-ß-catenin signaling pathways which control osteoclastogenesis and osteoblastogenesis, respectively. Alterations in their levels may influence bone remodeling both in inherited and acquired pediatric diseases (50–52). In another study, genetic variants ultimately lead to defects in hormonal signaling, paracrine signaling and extracellular matrix (48). Therefore, further studies of the specific mechanisms of altered bone metabolism in children with CP are needed in the above and other areas.

25-OHD is necessary for the maintenance of calcium homeostasis (53). As 25-OHD is the main storage form of vitamin D, its levels are used clinically to determine vitamin D status (54). Dietary intake and skin synthesis are two major sources of vitamin D (55). Our findings showed that the 25-OHD values of patients with CP were similar to those in the control groups, which is consistent with previous studies (56, 57). In addition, we observed the levels of 25-OHD decreased significantly with age. One possible reason is that exogenous supplementation of vitamin D is reduced in children older than 2 years (58).

PTH is the most important endocrine regulator of calcium and phosphorus concentration in extracellular fluid (59). It is secreted by the parathyroid gland in response to low Ca^2+^ concentrations of extracellular (60). The primary role of PTH is to increase tubular reabsorption of Ca^2+^ in the kidney and promote renal excretion of phosphate (61). Previous studies suggested that CP is associated with phosphorus-calcium metabolic disorders (62). Similarly, our study demonstrated that the level of PTH was significantly lower in the CP group at age 2.1–4. Future studies are needed to determine if appropriate treatment for phosphorus-calcium metabolic disorders can improve bone metabolism associated with CP.

In our study, concentrations of five indicators revealed no significant difference between GMFCS (I-III) and GMFCS (IV/V), spastic CP and non-spastic CP, or hemiplegia, diplegia and quadriplegia. Further, the exploratory analysis did not find compelling evidence that the relationship between the primary bone resorption marker, β-CTX, with the primary bone formation markers, TPINP and OC, differed between groups. Thus, given the higher y-intercept, bone formation may slightly outpace bone resorption in this cohort of children with CP as compared to healthy controls. However, these analyses were exploratory and should be interested as hypothesis-generating as opposed to confirmation of associations.

There were limitations in this present study. First, there was a limited sample size, especially for healthy group from years 0 to 2. Nevertheless, our findings in healthy groups are consistent with existing literature (28), suggesting the methods used in this study can be appropriate to make interpretations for the findings in children with CP. Second, the indicators of bone metabolism used in this study are typical and commonly used in clinical practice, but they do not fully capture the complexity of bone metabolism during development and for skeletally complex populations, like CP. Third, the mechanisms of abnormal bone metabolism in CP are not well understood, which may serve as a new direction for our future research.

## Conclusion

In summary, this study demonstrates that certain indicators of bone metabolism in children with CP differ from healthy controls at certain pre-pubertal ages. These findings may be helpful for understanding bone health and development in children with CP from the context of bone metabolism.

## Data Availability

The raw data supporting the conclusions of this article will be made available by the authors, without undue reservation.
