# Supplementary material for: Abnormal changes of bone metabolism markers with age in children with cerebral palsy
Source: Front Pediatr. 2023 Aug 1;11:1214608. doi: 10.3389/fped.2023.1214608 (PMC10427878; doi:10.3389/fped.2023.1214608)
Supplement: Supplementary file 1 [file Table1.docx]

**SUPPLEMENT TABLE 1** Baseline characteristics of spastic group and non-spastic group.

| clinical characteristics | Spastic  (n=57) |  | Non-Spastic  (n=18) |  | *p*-value |
| --- | --- | --- | --- | --- | --- |
| Age in years |  |  |  |  |  |
| - 0-2 | 0.95±0.120  19(33.3%) |  | 0.87±0.188  8(44.4%) |  | 0.191 |
| - 2.1-4 | 2.35±0.487  23(40.4%) |  | 2.20±0.447  5(27.8%) |  | 0.645 |
| - 4.1-7 | 4.80±0.862 |  | 4.60±0.894 |  | 0.749 |
|  | 15(26.3%) |  | 5(27.8%) |  |  |
| Gender, n (%) |  |  |  |  |  |
| - Male | 41(71.9%) |  | 8(44.4%) |  | 0.047 |
| - Female | 16(28.1%) |  | 10(55.6%) |  |  |
|  |  |  |  |  |  |
| CP Subtypes, n (%) |  |  |  |  |  |
| - Spastic hemiplegia | 16(28.1%) |  | 0(0.0%) |  |  |
| - Spastic diplegia | 32(56.1%) |  | 0(0.0%) |  |  |
| - Spastic quadriplegia | 9(15.8%) |  | 0(0.0%) |  |  |
| - Ataxic | 0(0.0%) |  | 2(11.1%) |  |  |
| - Dyskinetic | 0(0.0%) |  | 9(50.0%) |  |  |
| - Non-Classified | 0(0.0%) |  | 7(38.9%) |  |  |
|  |  |  |  |  |  |
| GMFCS level, n (%) |  |  |  |  |  |
| - I | 12(21.0%) |  | 0(0.0%) |  |  |
| - II | 17(29.8%) |  | 2(11.1%) |  |  |
| - III | 16(28.1%) |  | 3(16.7%) |  |  |
| - IV | 7(12.3%) |  | 7(38.9%) |  |  |
| - V | 5(8.8%) |  | 6(33.3%) |  |  |
|  |  |  |  |  |  |
| Complications, n (%) |  |  |  |  |  |
| - Cortical visual impairment | 8(14.0%) |  | 4(22.2%) |  |  |
| - Sensorineural auditory | 0(0.0%) |  | 1(5.6%) |  |  |
| impairment |  |  |  |  |  |
| - Communication difficulties | 14(24.6%) |  | 11(61.1%) |  |  |
| - Cognitive impairment | 11(19.3%) |  | 6(33.3%) |  |  |
| - Feeding difficulties | 1(1.8%) |  | 0(0%) |  |  |
| - Epilepsy | 8(14.0%)) |  | 3(16.7%) |  |  |
| Anti-convulsant treatments | 11(19.3%) |  | 4(22.2%) |  |  |

Data are shown as n (%) or mean± SD. CP, Cerebral palsy; GMFCS, Gross Motor Function Classification System.

**SUPPLEMENT TABLE 2** Baseline characteristics of GMFCS(I-III) group and GMFCS(IV-V) group.

| clinical characteristics | GMFCS(I-III) (n=50) |  | GMFCS(IV-V)  (n=25) |  | *p*-value |
| --- | --- | --- | --- | --- | --- |
| Age in years |  |  |  |  |  |
| - 0-2 | 0.96±0.094  13(26.0%) |  | 0.89±0.177  14(56.0%) |  | 0.256 |
| - 2.1-4 | 2.38±0.498  21(42.0%) |  | 2.14±0.378  7(28.0%) |  | 0.372 |
| - 4.1-7 | 4.88±0.885 |  | 4.25±0.500 |  | 0.333 |
|  | 16(32.0%) |  | 4(16.0%) |  |  |
| Gender, n (%) |  |  |  |  |  |
| - Male | 34(68.0%) |  | 15(60.0%) |  | 0.608 |
| - Female | 16(32.0%) |  | 10(40.0%) |  |  |
|  |  |  |  |  |  |
| CP Subtypes, n (%) |  |  |  |  |  |
| - Spastic hemiplegia | 15(30.0%) |  | 1(4.0%) |  |  |
| - Spastic diplegia | 27(54.0%) |  | 5(20.0%) |  |  |
| - Spastic quadriplegia | 3(6.0%) |  | 6(24.0%) |  |  |
| - Ataxic | 2(4.0%) |  | 0(0.0%) |  |  |
| - Dyskinetic | 2(4.0%) |  | 7(28.0%) |  |  |
| - Non-Classified | 1(2.0%) |  | 6(24.0%) |  |  |
|  |  |  |  |  |  |
| GMFCS level, n (%) |  |  |  |  |  |
| - I | 12(24.0%) |  | 0(0.0%) |  |  |
| - II | 19(38.0%) |  | 0(0.0%) |  |  |
| - III | 19(38.0%) |  | 0(0.0%) |  |  |
| - IV | 0(0.0%) |  | 14(56.0%) |  |  |
| - V | 0(0.0%) |  | 11(44.0%) |  |  |
|  |  |  |  |  |  |
| Complications, n (%) |  |  |  |  |  |
| - Cortical visual impairment | 9(18.0%) |  | 3(12.0%) |  |  |
| - Sensorineural auditory | 0(0.0%) |  | 1(4.0%) |  |  |
| impairment |  |  |  |  |  |
| - Communication difficulties | 13(26.0%) |  | 12(48.0%) |  |  |
| - Cognitive impairment | 9(18.0%) |  | 8(32.0%) |  |  |
| - Feeding difficulties | 0(0.0%) |  | 1(4.0%) |  |  |
| - Epileps | 6(12.0%)) |  | 5(20.0%) |  |  |
| Anti-convulsant treatments | 8(16.0%) |  | 7(28.0%) |  |  |

Data are shown as n (%) or mean± SD. CP, Cerebral palsy; GMFCS, Gross Motor Function Classification System.

**SUPPLEMENT TABLE 3** Baseline characteristics of spastic subtypes in CP group.

| clinical characteristics | Hemiplegia (n=16) | Diplegia  (n=32) | Quadriplegia  (n=9) | *p*-value |
| --- | --- | --- | --- | --- |
| Age in years |  |  |  |  |
| - 0-2 | 7(43.8%) | 8(25.0%) | 4(44.4%) |  |
| - 2.1-4 | 8(50.0%) | 14(43.8%) | 1(11.1%) | 0.290 |
| - 4.1-7 | 1(6.2%) | 10(31.2%) | 4(44.4%) |  |
|  |  |  |  |  |
| Gender, n (%) |  |  |  |  |
| - Male | 10(62.5%) | 25(78.1%) | 6(%) | 0.488 |
| - Female | 6(37.5%) | 7(21.9%) | 3(%) |  |
|  |  |  |  |  |
| GMFCS level, n (%) |  |  |  |  |
| - I | 7(43.7%) | 4(12.5%) | 1(66.7%) |  |
| - II | 7(43.7%) | 10(31.3%) | 0(0.0%) |  |
| - III | 1(6.3%) | 13(40.6%) | 2(22.2%) |  |
| - IV | 1(6.3%) | 4(12.5%) | 2(22.2%) |  |
| - V | 0(0%) | 1(3.1%) | 4(44.4%) |  |
|  |  |  |  |  |
| Complications, n (%) |  |  |  |  |
| - Cortical   visual impairment | 2(12.5%) | 4(12.5%) | 2(22.2%) |  |
| - Sensorineural auditory | 0(0.0%) | 0(0.0%) | 0(0.0%) |  |
| impairment |  |  |  |  |
| - Communication difficulties | 2(12.5%) | 7(21.9%) | 5(55.6%) |  |
| - Cognitive impairment | 0(0.0%) | 6(18.8%) | 5(55.6%) |  |
| - Feeding difficulties | 0(0.0%) | 0(0.0%) | 1(11.1%) |  |
| - Epilepsy | 3(18.8%) | 2(6.3%) | 3(33.3%) |  |
| Anti-convulsant treatments | 5(31.3%) | 2(6.3%) | 4(44.4%) |  |

Data are shown as n (%) or mean± SD. CP, Cerebral palsy; GMFCS, Gross Motor Function Classification System.
